# The immunomodulatory potential of bradykinin signaling in autoimmune conditions

**DOI:** 10.3389/fimmu.2026.1740800

**Published:** 2026-02-03

**Authors:** Magdalena Szaryńska, Agata Olejniczak-Kęder

**Affiliations:** Department of Histology, Medical University of Gdansk, Gdansk, Poland

**Keywords:** autoimmunity, BK inhibitor, bradykinin, IBD – inflammatory bowel disease, immunomodulation, rheumatoid arthritis, SLE – systemic lupus erythematosus

## Abstract

Bradykinin (BK) is a biologically active nanopeptide that plays a crucial role within the kallikrein–kinin system (KKS), a complex network involved in the regulation of vascular tone, epithelial cell ion transport, vascular permeability, mucosal secretion, release of cytokines from leukocytes among others. Over the past decades, BK has attracted sustained scientific interest due to its pleiotropic effects observed across various tissues and pathological conditions. Recent advances have significantly broadened our understanding of BK’s role in modulating inflammatory and immune processes. Notably, accumulating evidence indicates that BK can exert dual and context-dependent effects—either pro-inflammatory or anti-inflammatory—depending on the cellular environment, receptor subtype activation (BK1R vs BK2R), and crosstalk with other signaling pathways. Emerging studies highlight that BK receptors may interact with another surface molecules expressed on immune cells, including T cell receptors (TCR) and immune checkpoint proteins such as PD-L1. These interactions suggest that BK signaling may be in a center of crucial immunoregulatory mechanisms influencing leukocyte activation status. Such findings may have important implications for understanding immune homeostasis and for designing novel therapeutic strategies. In cancer, BK is suggested to contribute to tumor progression through the promotion of cancer stem cells and immunosuppressive microenvironment formation, whereas in autoimmune diseases, its modulation could attenuate excessive immune activation and tissue damage. Therefore, the dual nature of BK action positions it as both a potential therapeutic target and a modulatory agent depending on disease context. This review summarizes current knowledge on the multifaceted roles of BK in inflammation and immunity, emphasizing its molecular mechanisms, receptor dynamics, and potential therapeutic applications. Special attention is given to the interplay between BK signaling and regulatory membranous proteins, offering a framework for future research aimed at exploiting BK pathways to either suppress chronic inflammation or overcome tumor-associated immunosuppression.

## 1 Introduction

**Figure 1:**
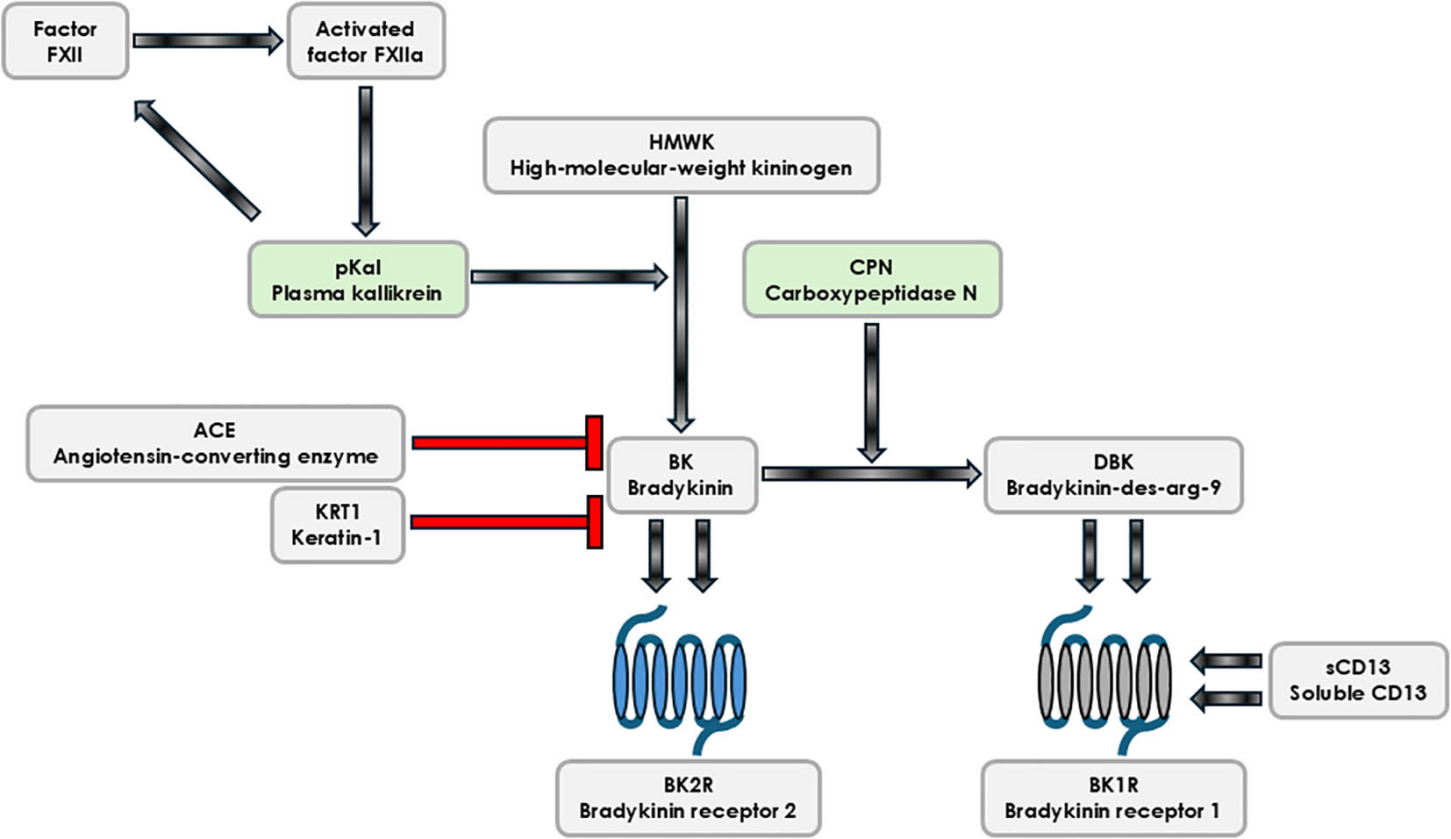
Bradykinin forming cascade. The kallikrein–kinin system (KKS) regulates vascular, inflammatory, and immune responses through the generation of bradykinin (BK) and its metabolite BK-des-arg-9 (DBK). Plasma kallikrein (pKal) cleaves high-molecular-weight kininogen (HMWK) to release BK, which is further processed by kininase I/carboxypeptidase N (CPN) to form DBK. BK and DBK mediate their effects via bradykinin receptor 2 (BK2R) and bradykinin receptor 1 (BK1R), respectively. Factor XII (FXII) circulates in a closed, inactive conformation bound to pKal and undergoes activation upon contact with surface domains, producing FXIIA (FXIIA – activated factor XII) through pKal-mediated proteolysis. FXIIA promotes additional BK release from HMWK, propagating KKS signaling. Soluble CD13 (sCD13), an ectopeptidase-derived pro-inflammatory mediator, acts via BK1R. BK is inactivated primarily by angiotensin-converting enzyme (ACE), linking KKS to the renin–angiotensin system, while keratin-1 (KRT1) inhibits BK expression. FXII, factor XII; FXIIA, activated factor XII; HMWK, high-molecular-weight kininogen; pKal, plasma kallikrein; BK, bradykinin; DBK, bradykinin-des-arg-9 (BK-des-arg-9); CPN, kininase I/carboxypeptidase N; BK1R, bradykinin receptor 1; BK2R, bradykinin receptor 2; ACE, Angiotensin-converting enzyme; sCD13, soluble CD13. Key enzymes in the KKS cascade have been highlighted in a green color on the figure.

Bradykinin (BK) is a linear nanopeptide belonging to the family of kinins, biologically active peptides generated through proteolytic processing of kininogens by kallikreins, a group of serine proteases. Plasma kallikrein cleaves high-molecular-weight kininogen (HMWK) (illustrated on **Figure 1**), primarily synthesized in the liver, to release BK, while tissue kallikreins act on low-molecular-weight kininogen (LMWK) to produce kallidin (kinins) (1, 2). These peptides constitute a key arm of the kallikrein–kinin system (KKS), which controls the vascular homeostasis and prevents pathological outcomes such as angioedema. Structurally, BK and its derivatives activity depends on rapid enzymatic processing and receptor engagement, giving them a central role in acute and chronic inflammatory responses. In next step, BK is acted upon by kininase I/carboxypeptidase N (CPN) to generate bradykinin-des-arg-9 (BK-des-arg-9) (DBK), an octapeptide (3, 4).

BK and DBK exert their biological effects through their receptors, bradykinin receptor 2 (BK2R) and bradykinin receptor 1 (BK1R), respectively (general characteristics of both receptors are summarized in **Table 1**). Both BK1R and BK2R bear seven helix transmembrane domains with a configuration shared with rhodopsin family of G protein coupled receptors (5). While BK2R is constitutively expressed in sensory nerve fibers, leukocytes, mast cells and endothelial cells (6), the expression of BK1R is very low in normal tissue unless stimulated by tissue injury, cytokines, LPS or inflammation (7). Upon tissue injury or exposure to cytokines like IL-1β and TNFα, the expression of BK1R is believed to be upregulated under the control of the MAP kinase pathway among others (8). The more, sequencing of the human BK1R has shown the existence of a transcriptional regulation site for NF-κB in the promoter region (9). BK1R is also reported to enhance the production of secondary mediators like prostanoids, tachykinins, nitric oxide and mast cell derived products, which help in propagating inflammatory and nociceptive processes (5). Additionally, BK2R regulates epithelial barrier function via ADAM17–EGFR transactivation, leading to rearrangement of tight junction proteins such as zonula occludens-1 (10). Interestingly, BK1R function can be downregulated by BK2R co-endocytosis and signaling suggesting new approaches to control BK1R pathway in some pathological conditions as was suggested based on the experiments conducted on kidney epithelial (human embryonic kidney HEK293 cells) and bovine pulmonary artery endothelial cells models (11).

**Table 1:** Biological features and the functions of BK1R and BK2R in physiological processes and in pathogenesis and progression of some autoimmune diseases.

BK2R Bradykinin receptor 2 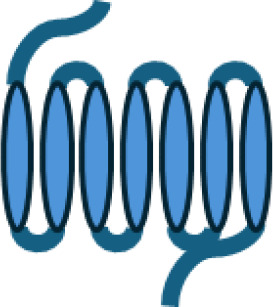	BK1R Bradykinin receptor 1 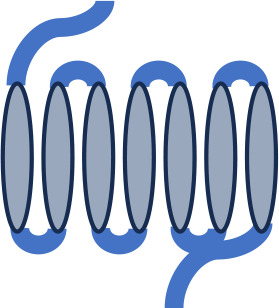
Physiological features and functions	
▪ Ligand: Bradykinin ▪ Constitutively present in sensory nerve fibers, mast cells and endothelial cells ▪ Expressed in major human leukocyte subsets, including T lymphocytes, monocytes and neutrophils, with monocytes exhibiting the highest receptor density ▪ Inflammation sustaining ▪ Contributes to acute inflammation and pain ▪ Involved in the initiation of adaptive immune responses ▪ drugs which target the BK2R: Icatibant, Anatibant, Fasitibant, Labradimil, Deltibant/Bradycor	▪ Ligands: Bradykinin-des-arg-9, soluble CD13 ▪ Expression is minimal under basal conditions ▪ Inducible by tissue injury, cytokines, LPS or inflammation ▪ Transcriptionally regulated by NF-κB ▪ Downregulated by BK2R co-endocytosis ▪ Resists desensitization ▪ Amplifies inflammation and vascular responses ▪ Enhances the production of secondary mediators like prostanoids, tachykinins, nitric oxide and mast cell derived products which help in propagating inflammatory and nociceptive processes ▪ drugs which target the BK1R: BAY 239584, Safotibant, SSR240612
Role in pathogenesis of autoimmune diseases	
▪ Sustains inflammation responsible for: ▪ Joint destruction in rheumatoid arthritis ▪ Kidney glomerular pathology in lupus nephritis ▪ Colitis ▪ Increases cyclooxygenase-2 (COX-2) – the key mediator of inflammation in inflammatory bowel disease ▪ Promotes production of pro-inflammatory cytokines ▪ Promotes leukocytes attraction and tissue infiltration ▪ Increases vascular leakage and permeability causing angioedema ▪ Responsible for intestinal barrier disruption via down regulation of zonula occludens structure ➔ compromising epithelial integrity and increasing intestinal permeability ▪ Contributes to secretory diarrhea	

Factor XII is a plasma protein closely associated with KKS system. It is found in its closed nctive form bound to pKal (12). Following the recognition of an activating surface domain, FXII alters conformation (shifts to its open structural conformation) and then is more susceptible to proteolytic cleavage by activated pKal, generating activated FXII (FXIIa). FXIIa is subsequently ready for next KKS system cascade steps, namely FXII acts on HMWK to release extra fraction of BK (13, 14) what contributes to increased BK availability in local niche.

Given their potent vasoactive and proinflammatory properties, kinins require strict regulation through enzymatic degradation. Angiotensin-converting enzyme (ACE) is the primary enzyme responsible for BK inactivation, thus linking KKS with the renin–angiotensin system (15). ACE inhibitors, widely used as antihypertensive drugs, can inadvertently elevate BK levels, occasionally leading to drug-induced angioedema (16). In contrast, ACE2 preferentially degrades DBK derivatives, thereby modulating BK1R signaling in the lung and offering protection against pulmonary edema in inflammatory states (17). BK1R interaction with angiotensin type I receptor (AT1R) was clearly evidenced on mouse model of angiotensin II – induced hypertension (18). Disruption of this balance, as seen in viral infections such as SARS-CoV-2, where ACE2 function is downregulated, has been hypothesized to exacerbate local pulmonary inflammation and angioedema (19–22).

Beyond their canonical vascular effects, BK and its receptors have been implicated in diverse pathological processes, including inflammation (1), cancer (1, 23), pain (24) and cardiovascular diseases (25). The more, upregulation of BK receptors is associated with the onset of some autoimmune diseases what was demonstrated in animal models with deficiency of BK1R/BK2R or with the use of specific inhibitors/antagonists, like it was mentioned in psoriasis (26), autoimmune central nervous system disease (27), asthma DCs (28), experimentally induced nephritis (29) and lupus (7).

Genetic deletion of KKS components or BK receptors in animal models confers protection against experimental inflammatory diseases, highlighting the pathogenic role of excessive kinin signaling (30–33). Consequently, BK represents not only a central mediator of vascular physiology but also a critical driver of inflammatory pathology, making its signaling axis an attractive therapeutic target across multiple clinical contexts. Peptidic and non-peptidic receptor antagonists (such as Icatibant, Anatibant, Bradycor, and Labradimil) have been developed as potential therapeutic agents and some of these have progressed to clinical trials, as summarized by Wiśniewski P. et al. (34) and presented by others (35–38). An analysis of the properties of BK and its receptors is essential for gaining an in-depth understanding of disease pathogenesis, with particular emphasis on the role of BK in the development of autoimmune disorders. The growing clinical importance of this research is underscored by large-scale epidemiological data: an analysis of anonymized electronic health records from 22 million individuals in the United Kingdom revealed that autoimmune diseases affect approximately 10% of the population, with their prevalence continuing to rise over time at varying rates across specific disorders (39). This finding provides a striking illustration of the increasing burden of autoimmune diseases and highlights their growing significance as a pressing challenge for public health systems worldwide. A more detailed description of selected autoimmune diseases, which represent a major clinical challenge, is presented in the subsequent sections of this work.

## 2 The bradykinin pathway for immune cell function

Flow cytometric analyses demonstrated that BK2R is expressed across major human leukocyte subsets, including T lymphocytes (CD3+), monocytes (CD14+), and neutrophils (CD16b+), with monocytes exhibiting the highest receptor density (6, 28). Functional engagement of BK2R in monocytes is confirmed by phosphorylation of Erk2, consistent with downstream signaling activation. In contrast, BK1R expression is minimal under basal conditions, but is strongly induced in leukocytes upon exposure to pro-inflammatory stimuli such as lipopolysaccharide (LPS), cytokines, and tissue damage. This inducible pattern suggests that BK1R plays a specialized role in amplifying inflammation.

### 2.1 The functions of BK1R and BK2R in immune regulation

Although BK2R is constitutively expressed on the surface of various leukocyte populations, its role in the initiation and progression of inflammatory responses is indispensable. BK2R contributes to the maintenance of inflammatory processes by sustaining monocyte and lymphocyte activation (40, 41). Its role in monocyte recruitment and maturation has been also proposed (6). In murine models of autoimmune encephalomyelitis, monocyte BK2R signaling regulates chemokine expression and leukocyte trafficking within cerebral microvasculature (42). Recent studies have demonstrated enhanced adhesion properties associated with BK signaling, both during the *Plasmodium falciparum* erythrocytic cycle (43) and in glioblastoma patients (44). Furthermore, pharmacological inhibition of BK receptors, which is known to confer cardioprotective effects, has been proposed as a potential therapeutic strategy in a murine model of Chagas disease cardiomyopathy (45). Flow cytometric analysis (FACS) revealed that the frequency of proinflammatory monocytes was reduced in BK1R^-^/^-^ hearts, whereas elevated creatine kinase activity at 60 days post-infection was observed exclusively in BK1R^+^/^+^ sera. Additionally, increased expression of BK receptors has been reported in patients experiencing the acute phase of hereditary angioedema (46).

Beyond innate functions, BK2R is also implicated in adaptive immune priming. During *Trypanosoma cruzi* infection, dendritic cells (DCs) sense parasite-derived kinins via BK2R, which is essential for the generation of IFN-γ–producing effector T cells. BK2R-deficient mice show impaired T cell responses, a defect rescued by transfer of wild-type DCs, thereby directly linking BK2R signaling to antigen presentation and protective immunity (47). Furthermore, it was indicated that kinin peptides can serve as danger signals that trigger DCs to produce IL-12 through activation of BK2R that drives Th1 polarization (47, 48). The more, the trypomastigotes (the extracellular infective forms of trypanosomes) failed to up-regulate type 1 immunity in TLR2(-/-) mouse model of subcutaneous infection by Trypanosoma cruzi. That observation suggests the cooperative activation of TLR2 and BK2R for induction of type 1 immunity (47). This finding appears to have far greater significance than initially expected. BK and its receptors are likely to exert a direct influence on leukocyte functions under various pathological conditions. A detailed understanding of these interactions may help to elucidate the true relevance of these phenomena for the effectiveness of therapeutic strategies in patients suffering from autoimmune diseases, infections, or malignancies.

BK1R signaling contributes to vascular leakage, vasodilation, and leukocyte recruitment during inflammation. BK1R via DBK enhances neutrophil attraction and tissue infiltration, consistent with its role in acute inflammation. Recent investigations, conducted both *in vivo* and *in vitro*, indicate that stimulating BK1R in neutrophils during inflammation promotes their attachment and movement to nearby tissues. The interaction of DBK with BK1R leads to heightened vascular permeability (49) enhanced neutrophil attraction, and triggers broncho- and vasoconstriction, sparking inflammation (50).

A critical immune-related pathway involves the ectopeptidase CD13, which can be shed as soluble CD13 (sCD13) compound. sCD13 acts as a potent pro-inflammatory mediator through BK1R, inducing chemotaxis, angiogenesis, and phosphorylation of Erk1/2. These effects are abolished by BK1R antagonists, identifying BK1R as the key receptor mediating the arthritogenic and angiogenic actions of sCD13. In viral infection, elevated sCD13 levels were observed in patients with severe COVID-19, correlating with inflammation, neutrophil extracellular trap (NET) formation, and poor outcomes (51). Neutrophil responses to sCD13, including chemotaxis and NETosis, are significantly reduced upon BK1R blockade, underscoring its role in neutrophil-driven hyperinflammation.

Although both BK receptors are activated by their ligands in patients infected with SARS-CoV-2, one study suggested that blockade of the BK2R and inhibition of pKal activity may have particularly beneficial effects in these patients. Such interventions may exert a mitigating effect in the early stages of COVID-19 and potentially prevent the development of acute respiratory distress syndrome (ARDS). Furthermore, anti-inflammatory agents can also indirectly modulate this pathway, as local pro-inflammatory cytokines are known to induce BK-dependent effects (50). Inhibition of NF-κB translocation, TNFα, or IL-1 has been shown to prevent the upregulation of the BK1R-mediated signaling pathway induced by LPS (52). However, these strategies may also contribute to certain systemic side effects, which should be carefully considered when planning treatment. All proposed anti-inflammatory strategies should be implemented alongside blockade of the KKS and available targeted therapies (such as antiviral drugs used in COVID-19 patients) as early as possible during disease progression (53). Such anti-inflammatory approaches may significantly prolong the therapeutic window available to patients.

Moreover, recent findings from a glioblastoma (GBM) model highlight the importance of the interactions between distinct lymphocyte membrane proteins and BK receptors. Specifically, BK1R was shown to induce the expression of the immune checkpoint protein PD-L1 in GBM cells and macrophages, thereby enabling them to escape attacks from immune cells (44). Although these experiments were conducted within the tumor microenvironment—where the progressive increase in immunosuppression creates a distinct immunological context—the observation that BK can modulate immune checkpoint proteins remains highly significant. It suggests that BK may induce both proinflammatory and tolerogenic responses in leukocytes. This dual potential highlights the importance of exploring novel hypotheses regarding the therapeutic modulation of BK signaling in autoimmune diseases. Indeed, not only blocking BK pathways but also selectively stimulating BK receptors under tightly defined conditions may yield substantial clinical benefits.

### 2.2 The mast cell–bradykinin axis

Mast cells are known to be a population largely responsible for the development of immune cell hyperreactivity across various organs and under a wide range of pathological events. That is the reason why these cells remain under continuous investigation in the context of autoimmune disease development.

Mast cell degranulation may promote the KKS cascade, leading to BK generation through a heparin-dependent pathway. Heparin released from mast cells, owing to its relatively high degree of sulfation, is capable of initiating the BK-forming cascade by activation of factor XII (FXII), which subsequently triggers pKal functions and BK release (54, 55). This pathway enhances vascular permeability and contributes to angioedema, hypotension, and airway obstruction in allergic and anaphylactic reactions. *In vitro* experiments with human plasma and animal models, and finally clinical observations in patients suffering from hereditary angioedema due to C1 inhibitor deficiency (HAE-C1-INH) served as models used to investigate the above-mentioned function of the KKS and BK systems. BK is supposed to be an explanation of the higher incidence rate of another hypersensitivity reactions in these patients. The manifestations of hypersensitivity reactions favor angioedema attacks, and therefore HAE-C1-INH is more easily diagnosed in these patients than in C1-inhibitor deficient patients without hypersensitivity reactions (56). Scientific evidence summarized in (57) strongly suggests that hypersensitivity reactions induced by commonly occurring environmental allergens are closely associated with elevated BK levels in the population of patients with HAE-C1-INH. Horvath et al. (56) reported that hypersensitivity reactions of other etiologies occur approximately three times more frequently in these patients. At the same time, it should be emphasized that a comprehensive understanding of these relationships requires further extensive investigation.

Additionally, noteworthy observations indicate the existence of an alternative mechanism triggering mast cell degranulation that is independent of the IgE receptor. It has been demonstrated that Mas-related G protein-coupled receptor-X2 (MRGPRX2) may serve as a target for its endogenous pro-inflammatory ligands as well as for diverse environmental compounds (58).

### 2.3 Pathophysiological consequences and therapeutic implications

It is well known that excess of BK production leads to vasodilation, vascular permeability, and hypotension, contributing to conditions such as angioedema, cardiovascular dysfunction and immune hyperactivity. Dysregulated BK1R and BK2R signaling amplifies immune cell recruitment and activation, thereby exacerbating inflammatory pathology in autoimmunity, infection, and allergy. Therapeutically, BK receptor antagonists (e.g. Icatibant), monoclonal antibodies, and complement inhibitors are emerging as promising strategies to counteract BK-mediated inflammation (59–62). Such interventions have already shown benefits in hereditary angioedema and hyperactivation of immune cells (including mast cells); thus they are being investigated in other inflammatory diseases. The other study confirmed that both the BK1R and BK2R antagonists can alleviate virus-induced ARDS in COVID-19 patients (50) what creates tremendous opportunities to enhance the effectiveness of therapy for patients with COVID-19 infection. Additionally, Icatibant is suggested to give some improvement for Puumala hantavirus infected patients (63). Additionally, pharmacologic inhibition of factor XII, pKal, HMWK, or the BK2R, but not the BK1R, largely attenuated allergen/IgE-mediated mast cell hyperresponsiveness in mice. The severity of anaphylaxis was associated with mast cell degranulation, increased plasma heparin levels, the intensity of KKS activation, and BK formation (60). The more, the differential expression of KKS proteins and receptors on peripheral blood neutrophils emerged as a promising biomarker for arthritis (64), providing a minimally invasive and effective mean to assess inflammatory activity in arthritis. Such differences in KKS expression may also serve as indicators of pain intensity and responsiveness to BK1R-targeted therapies (64).

## 3 The role of bradykinin in inflammatory bowel disease

Inflammatory Bowel Diseases (IBD) is a group of chronic autoimmune gastrointestinal inflammatory conditions with a continuously increasing incidence rate that can involve the entire gastrointestinal tract (65, 66). IBD affects up to 1% of the population constituting a serious clinical problem (67, 68). IBD is clinically classified as ulcerative colitis (UC) or Crohn’s disease (CD). The location of the inflammation, disease’s behavior, symptoms and histological features differentiate between the two diseases. The interplay between several factors, such as the intestinal microbiota, immune system, genetic predisposition, and environment affect the onset and development of IBD (65, 69).

KKS system has emerged as a significant contributor to the pathogenesis of IBD. Experimental colitis models have demonstrated that genetic deficiency of HMWK, pKal or BK receptors results in attenuated disease severity, including reduced body weight loss, disease activity index, colon shortening, histological damage, and colonic cytokine production. Reconstitution of HMWK-deficient mice restored BK release, IL-1β expression, and susceptibility to colitis, further implicating BK as a mediator of intestinal inflammation (30).

BK1R is minimally expressed under physiological conditions but is strongly upregulated in inflamed intestinal tissue, partly under the control of TNFα (70). Unlike BK2R, BK1R resists desensitization, thereby sustaining proinflammatory signaling during persistent disease activity. At the same time, an experimental study examined the involvement of BK1R in a TNBS (2,4,6-trinitrobenzene sulfonic acid)-induced mouse model of colitis, demonstrating that the selective, orally active, non-peptide BK1R antagonist SSR240612 significantly attenuated TNBS - induced colitis, including intestinal tissue injury and neutrophil infiltration (71). The more, both receptors promote cytokine production, including IL-1β, IL-6, and TNFα, as well as leukocyte infiltration into the colonic mucosa, amplifying the inflammatory cascade (70).

Recent investigations have demonstrated that BK possesses the capacity to modulate the properties of zonula occludens junctions (tight junctions), the proper function of which is required for preserve intestinal epithelial integrity in IBD patients. BK2R regulates epithelial barrier function via ADAM17–EGFR transactivation, leading to rearrangement of tight junction proteins such as zonula occludens-1 (10). Since keratin-1 (KRT1) inhibits BK expression, the inhibition of KRT1 caused the downregulation of tight junctions proteins (such as occludin, zonula occludens-1 and claudin) what suggested the close association between these cellular proteins (72). This process compromises epithelial integrity and increases intestinal permeability, a hallmark feature of ulcerative colitis and Crohn’s disease. By inhibiting BK expression and inflammatory cytokines while promoting intestinal barrier markers, KRT1 helps prevent colonic damage and sustain barrier integrity. In addition, both BK1R and BK2R mediate ion transport responses in the intestinal epithelium, contributing to secretory diarrhea (70).

Collectively, these data suggest that BK acts as a critical mediator linking barrier dysfunction to mucosal immune activation in IBD. In particular, BK1R upregulation in the inflamed intestine provides a structural and functional basis for sustained kinin activity, highlighting selective BK1R antagonism as a novel and potentially effective therapeutic strategy in patients with refractory IBD.

Given these findings, BK pathway represents a promising therapeutic target in IBD. Pharmacological blockade of BK1R, either through selective antagonists (e.g., SSR240612) (31) or genetic ablation, has been shown to reduce neutrophil influx, lower cytokine levels, and improve macroscopic and histological indices of colitis in murine models (30). BK2R antagonism, while reducing intestinal motility and barrier alterations, appears to confer limited benefit in controlling inflammation. Additional strategies aimed at modulating KKS activity, including kallikrein inhibitors, kallistatin supplementation, or regulation of KRT1, also demonstrate potential in restoring barrier function and dampening intestinal inflammation (72). Additionally, infiltration of neutrophils and inflammatory monocytes in the colonic lamina propria was reduced in HMWK-deficient mice. Reconstitution of HMWK-deficient mice through intravenous injection of HMWK recovered their susceptibility to Dextran Sodium Sulfate (DSS)-induced colitis, increased IL-1β levels in the colon tissue and BK concentrations in plasma (30).

An intriguing finding emerging from recent research is the link between BK receptor–mediated signaling and the regulation of cyclooxygenase-2 (COX-2) activity (73). COX-2, an inducible isoform of cyclooxygenase, plays a central role in converting arachidonic acid into prostaglandins, key mediators of inflammation and tumor progression. The discovery that aspirin, the prototypical nonsteroidal anti-inflammatory drug (NSAID), inhibits COX-2 activity remains one of the principal explanations for the anticancer effects of NSAIDs, particularly through the reduction of prostaglandin synthesis (74). Proinflammatory arachidonic acid derivatives have been consistently implicated in the progression of various malignancies, including colorectal cancer (CRC) (73). Taken together, the findings suggest that BK and its receptors remain the crucial paracrine mediator of cell-cell crosstalk in the setting of colitis-associated cancer.

Extensive evidence supports a critical role of COX-2 in the pathophysiology of CRC. COX-2 is strongly induced in the intestinal stroma of patients with IBD, and its activity has been implicated in all stages of colorectal carcinogenesis, ranging from adenoma formation to malignant progression (75). Both selective and non-selective COX-2 inhibitors have demonstrated clinical benefits in preventing and treating CRC, highlighting the importance of this pathway in tumor biology. Notably, TNFα has been shown to amplify BK-mediated protein kinase D (PKD) phosphorylation, a signaling event that results in synergistic induction of COX-2 expression in primary mouse and human colonic myofibroblasts (73). This mechanistic link suggests that BK signaling can intersect with cytokine-driven pathways to enhance COX-2 activity and thereby promote a proinflammatory and pro-tumorigenic microenvironment. Together, these findings indicate that the interplay between BK receptors and COX-2 may represent a critical axis in the pathogenesis of inflammation-associated CRC and could offer novel opportunities for therapeutic intervention.

Accumulating evidence highlights BK as a pivotal mediator in the pathogenesis of IBD, acting at the intersection of epithelial barrier dysfunction, cytokine-driven inflammation, and leukocyte recruitment. While both BK1R and BK2R contribute to disease progression, the inducible and non-desensitizing nature of BK1R signaling appears particularly relevant to the chronicity of IBD. The therapeutic efficacy of selective BK1R antagonists in preclinical models underscores the translational potential of targeting this pathway in patients who remain refractory to conventional therapies, including anti-TNF agents.

Future research should focus on clarifying the differential contributions of BK1R and BK2R in distinct clinical phenotypes of IBD, as well as investigating potential synergistic effects of BK pathway inhibition with existing biological properties. In addition, further studies are warranted to define the impact of pKal inhibitors, kallistatin, and KRT1 modulators on mucosal healing and long-term disease outcomes. Ultimately, therapeutic strategies directed at the BK axis may represent a novel and promising addition to the therapeutic strategies for IBD management.

## 4 The bradykinin pathway in systemic lupus erythematosus

Systemic lupus erythematosus (SLE) is a chronic autoimmune disease that causes inflammation and subsequent damage to a range of organs including skin, joints, and other major organs such as the kidneys, brain, lungs and heart. The course of SLE is typically characterized by periods of relapse and remission following treatment with immunosuppressive therapies (76). SLE can present at any age, with 15–20% of patients developing it during childhood. Adult women are nine times more likely to be affected than men, with a peak incidence in the reproductive years (77). Lupus nephritis (LN) is one of the commonest and most severe clinical features of SLE and leads to significant morbidity and mortality (78).

The exact etiology of SLE remains unknown, although combinations of genetic, environmental, immunological and hormonal factors appear to play a key role (79). The clinical presentation is widely variable including cardiovascular emergencies (pericarditis, cardiac tamponade, myocardial infarction, thrombosis), neurological (stroke), pulmonary (pulmonary hemorrhage/edema), and even renal (lupus nephritis; LN) (80). Since mortality in SLE patients is approximately five times higher than in the general population, it is very important to timely identify SLE in its specific form, and to initiate the appropriate treatment without delay.

KKS through generation of BK and DBK has emerged as an important modulator of systemic inflammation and autoimmunity in SLE (3, 5). Elevated levels of circulating DBK have been identified in both lupus-prone mice and patients with SLE, reaching over 100-fold and 22-fold higher concentrations than in healthy controls, respectively (7). These findings are unlikely to be the consequence of the drugs the patients were taking (e.g. ACE inhibitors) since similar findings were noted in murine model of lupus. This shift toward enhanced BK metabolism and preferential BK1R activation is thought to amplify pro-inflammatory cascades, including prostanoid release, nitric oxide generation, and chemokine-driven immune cell recruitment, thereby contributing to systemic autoimmunity and tissue injury (5, 9).

In LN, BK1R upregulation has been demonstrated in renal tissue, where its blockade ameliorates glomerular pathology, reduces proteinuria, and dampens local chemokine (CCL2, CCL5) expression as well as immune cell infiltration (78). This suggests that BK1R signaling plays a pivotal role in sustaining renal inflammation and could represent a therapeutic target, particularly in LN patients with concomitant hypertension. It has been shown that BK1R antagonism or ablation plays a protective role in nephrotoxic serum-induced glomerulonephritis (29), LPS-mediated acute renal inflammation (81), and experimental obstructive nephropathy (82).

Furthermore, studies have highlighted the capacity of kallikrein-1 (Klk1) – the another element of KKS supplementation to attenuate LN and neuropsychiatric lupus manifestations (83, 84) by reducing type I interferon responses, reduced plasma IFNα levels and proinflammatory cytokines, microglial activation, and depressive-like behavior in MRL/lpr mice (lupus prone mice model) (85). The more, Klk1 reduced IFNAR1 and JAK1 protein expression, important upstream molecules in Type I IFN signaling and reduced BK1R expression (85). Given that excessive interferon signaling is a hallmark of SLE, modulation of the KKS may provide dual benefits in both systemic and neuropsychiatric disease domains.

The clinical relevance of these observations is further supported by reports of acquired angioedema (AAE) in SLE patients, a condition linked to dysregulated BK production and C1 esterase inhibitor (C1-INH) deficiency, which can manifest as life-threatening laryngeal edema (86, 87). C1-INH is an important regulator of variable physiological processes, like complement activation, coagulation, fibrinolytic systems but also of the KKS. The swellings are caused by increased production of BK, resulting from activation of the KKS system (88). The analyses revealed that NK cell counts were lower in hereditary angioedema patients with deficiency of C1-INH (89). Additionally, Th cell balance was skewed towards more Th2 cells and less Th1 cells in those patients compared to controls. There were also lower frequencies of class-switched B cells and plasma cells in patients. Levels of C4 and the complement activation fragment C3d were related to disease activity (89).

All these findings and clinical observations highlight the broader implications of aberrant BK signaling in lupus pathogenesis, extending beyond nephritis and neuroinflammation to vascular and complement-mediated complications. Taken together, accumulating evidence suggests that BK and its receptors, particularly BK1R, function not only as biomarkers of disease activity but also as active drivers of inflammation, autoimmunity, and organ damage in SLE, underscoring their potential as therapeutic targets.

## 5 The bradykinin pathway in rheumatoid arthritis

BK and its receptors are critical mediators in the inflammatory pathways that contribute to arthritis, particularly rheumatoid arthritis (RA) pathogenesis. Elevated levels of BK and pKal as well as increased expression of the BK receptors were detected in the synovial fluid of arthritic patients (90). The more, the engagement of KKS system with BK as a crucial player in the progression of RA was reported in different experimental models (32, 51, 91). Genetic deletion of key KKS components, including pKal, HMWK, or BK receptors, confers protection against arthritis in mice, characterized by reduced joint swelling, diminished cytokine production, and decreased vascular permeability. These findings highlight the pathogenic relevance of BK generation in joint inflammation (33).

BK receptors are believed to be pivotal regulators of both acute and chronic phases of arthritis. The diverse expression of both BK receptors underlies their distinct functions in arthritis pathogenesis. BK2R primarily contributes to acute inflammation and pain, whereas BK1R is strongly implicated in chronic synovial inflammation, immune cell recruitment, and joint remodeling/destruction (32). In *ex vivo* RA synovial tissue organ cultures, a BK1R antagonist reduced secretion of inflammatory cytokines (1, 32, 92, 93).

The CD13–BK1R axis has recently been identified as a critical pathway driving RA pathogenesis. CD13, an aminopeptidase expressed on myeloid cells and fibroblast-like synoviocytes, can be shed into the extracellular space as soluble CD13 (sCD13). High concentrations of sCD13 are found in RA synovial fluid, where it exhibits potent pro-inflammatory and arthritogenic properties (94). Importantly, sCD13 engages BK1R to induce monocyte and neutrophil chemotaxis, robust phosphorylation of Erk1/2, angiogenesis, and cytokine secretion (32). These effects are independent of CD13 enzymatic activity, underscoring its function as a signaling ligand rather than an enzyme in this context. Mice lacking either CD13 or BK1R due to genetic deletion are protected against the development of inflammatory arthritis across several murine models (32). Pharmacological blockade of BK1R similarly reduces synovial inflammation and cytokine production, establishing BK1R as a key mediator of sCD13-driven arthritogenic responses (95).

Evidence also supports a direct role of BK in immune cell activation within arthritic joints. BK promotes monocyte activation, enhances the expression of pro-inflammatory receptors such as FcγRIII and C5aR, and amplifies immune complex–mediated KKS activation via pKal increased activity. All these effects induced elevated cytokine release and perpetuation of joint swelling and synovial infiltration (33). Moreover, neutrophils and monocytes contribute to sustaining local kinin production in the synovial environment, further fueling a cycle of inflammation (33, 64). Comparative studies across arthritis subtypes have shown higher expression of BK1R on circulating neutrophils from RA and gout patients compared with osteoarthritis, whereas BK2R expression appears more stable across disease groups (96).

From a therapeutic perspective, BK receptors blockade may attenuate arthritis; nevertheless, the differential expression and characteristics of these two receptors could lead to divergent outcomes (97). BK2R antagonists have shown efficacy in alleviating pain, but with limited benefit in reducing synovitis (92). In contrast, BK1R blockade demonstrates significant anti-inflammatory and disease-modifying potential in preclinical models (32, 95). Given its restricted expression in normal tissues and robust upregulation in inflamed joints, BK1R represents a particularly attractive therapeutic target for RA and potentially other forms of inflammatory arthritis. The study conducted on rat model of RA revealed increased plasma levels of BK following being insulted with streptococcal cell wall polymers, which led to polyarthritis (91). The inhibition of KKS in rat arthritis models minimized joint disability and reduced BK plasma levels (98, 99).

Given the current understanding that BK1R is involved in nociceptor activation, whereas BK2R contributes to their sensitization (64), the potential use of BK1R or BK2R inhibitors to pain relief requires further investigation before being implemented routinely in patients with RA and osteoarthritis (OA). The findings of various studies analyzing these mechanisms remain inconsistent. Plasma inflammatory biomarkers and BK1R expression on blood neutrophils correlated with pain as demonstrated in OA and RA patients (64). BK1R antagonism improves nociceptive tolerance and prevent joint cartilage degradation in a rat surgical model of osteoarthritis (93). Although BK2R antagonists (Icatibant) have demonstrated potential in alleviating pain in knee osteoarthritis, it rather seems that their constitutive expression and involvement in acute inflammation may restrict their effectiveness in chronic inflammatory conditions (92). In this study, the administration of BK2R antagonists resulted in significant improvements in pain both at rest and during activity, demonstrating their potential analgesic efficacy; however, no changes were observed in synovitis as assessed by contrast-enhanced ultrasound (92).

## 6 The role of bradykinin in cancer

The crucial role of KKS for tumor progression was evidenced by numerous *in vitro* and *in vivo* studies. The expression of BK receptors was demonstrated on CRC (23, 100), esophageal squamous cell carcinoma (101), breast cancer (102), head and neck squamous cell carcinoma (103) and prostate cancer (104, 105). The inducible BK1R receptor has received particular attention due to its variable roles across different cancer types. In some contexts, such as breast and prostate cancer, the direct stimulation of BK1R has been shown to function as a tumor promoter and modulator. The treatment of BK1R induced significant proliferation of MCF-7 breast cancer cells even at very low concentrations of ligand. That effect depended on the co-stimulation of EGFR and subsequent ERK1/2 phosphorylation (102). Similar effects have been observed following the BK1R agonists incubation in androgen-insensitive prostate cancer PC3 cells (104) and DU145 prostate cancer cells (105).

Inhibitors or antagonists of BK receptors have been proposed as potential therapeutic agents in cancer treatment. Studies have shown that administering BK inhibitors to various cancer cell types, both *in vitro* and *in vivo*, leads to a reduction in tumor mass, decreased aggressiveness, and diminished vascularization. Additionally, these treatments were associated with lower tumor cell proliferation and enhanced anti-tumor immune activity. Such effects have been observed in several cancer models, including melanoma (106), colorectal cancer (100), and glioblastoma (107). Interestingly, treatment of glioblastoma with agonists of either BK1R or BK2R produced no significant changes in tumor parameters in mouse model (107).

Additionally, BK and its derivatives exert profound effects on the regulation of cancer stemness and growth. Recent work demonstrated that chemically modified BK analogues can significantly reduce colonosphere size of CRC HCT116 cell line, while also lowering the proportion of CD133^+^ cancer stem cells (CSCs) (23). These findings are especially relevant, as CSCs are considered critical drivers of tumor initiation, resistance, and relapse (108). The observed reduction in CSC populations suggests that BK analogues could impair tumor propagation by disrupting stem cell–like features, highlighting their potential as lead compounds for novel anticancer therapeutics.

The more, in glioblastoma (GBM) the elevated BK1R expression was suggested to enhance malignancy. The more, an immunohistochemistry analysis of tumor samples from 16 patients with glioma diagnosis revealed a marked BK1R immunopositivity in low-grade gliomas or in older glioblastoma individuals (107). Further analysis presented that BK1R overexpression (using BK1R wild-type plasmid and *in vitro* BK1R overexpression (OE) model) (44) upregulates adhesion molecules (ICAM-1, VCAM-1), thereby promoting tumor cell migration and monocyte infiltration. In parallel, BK1R drives the production of pro-tumorigenic cytokines and chemokines (IL-6, IL-8, CXCL11, CCL5), fostering an immunosuppressive and growth-supportive tumor microenvironment (44). Moreover, BK1R OE confers resistance to oxidative stress through induction of the antioxidant protein HO-1 and facilitates immune evasion by stimulating PD-L1 expression in both GBM cells and tumor-associated macrophages (TAMs) (44). These findings clearly position BK1R as a key regulator of tumor aggressiveness and immune escape in gliomas.

The capacity of BK to influence immune regulation extends beyond tumor cell–intrinsic pathways. In GBM, BK1R overexpression promotes TAMs polarization, further amplifying immunosuppressive signaling in the tumor microenvironment (44). Such mechanisms underscore a dual role of BK signaling: on one hand, fostering proinflammatory and migratory responses; on the other, inducing tolerance-associated pathways, such as PD-L1 upregulation. This duality highlights the complexity of targeting BK signaling in cancer, where therapeutic approaches may need to account for both its tumor-promoting and immune-modulating functions.

The therapeutic exploitation of BK pathways has recently expanded to innovative drug delivery strategies. A BK-potentiating peptide–paclitaxel conjugate (BPP-PTX) has been engineered to selectively target angiotensin-converting enzyme (ACE), which is aberrantly expressed in triple-negative breast cancer (TNBC) cells but not in receptor-positive breast cancer or healthy tissues (109). This conjugate not only demonstrated improved tumor suppression compared to unmodified paclitaxel, but also showed reduced systemic toxicity, including less body weight loss and myelosuppression.

Of particular note is the observation that the conjugate, by inhibiting ACE activity, simultaneously suppresses the enzyme’s capacity to degrade BK within the tumor niche. This finding requires further investigation to determine the actual significance of the increased BK concentration during such therapeutic interventions. The context-dependent nature of BK signaling, however, remains a challenge, necessitating further mechanistic and translational studies to determine when BK acts as a friend or foe in cancer. Ultimately, unraveling these complexities could establish BK pathways as both biomarkers and therapeutic targets across a spectrum of malignancies.

## 7 Conclusions and perspectives

The BK receptors were initially defined in the late 1970s and 1980s (110) and originally associated with their impact on smooth muscle contraction, epithelial cell ion transport, vascular permeability, mucosal secretion, release of cytokines from leukocytes and sensory neurons (111). Recent studies, however, indicate that BK associated pathways may also exert immunomodulatory effects on cells across various organs. It has been suggested that BK signaling could serve as potential tool or therapeutic target for a range of diseases, including cancers and autoimmune disorders (34). In both cases, the immune system escapes the mechanisms controlling immune cell function and the inappropriate activation status is triggered, molecular effects induced by both BK1R and BK2R could help identify intracellular intersections with other signaling cascades that regulate the activity of specific leukocyte populations. Of particular interest is the potential to modulate the function of membrane receptors crucial for the activation of lymphocytes and antigen-presenting cells. Inhibitors with diverse chemical properties are currently being analyzed in both *in vitro* and animal models with the aim of potential inclusion in therapies for patients exhibiting dysregulated immune cell activity. Recently, some of these compounds have progressed to clinical trials. Such intensified research efforts offer hope for the development of a pool of drugs sharing immunomodulatory potential mediated through the BK pathway. Most importantly, observations concerning the BK pathway suggest that it has the dual nature what positions it as both a potential therapeutic target and a modulatory agent depending on disease context.
